# Publisher Correction: Cable bacteria reduce methane emissions from rice-vegetated soils

**DOI:** 10.1038/s41467-022-29008-x

**Published:** 2022-03-08

**Authors:** Vincent V. Scholz, Rainer U. Meckenstock, Lars Peter Nielsen, Nils Risgaard-Petersen

**Affiliations:** 1 Center for Electromicrobiology, Aarhus University, 8000 Aarhus C, Denmark; 2 Environmental Microbiology and Biotechnology, University Duisburg-Essen, 45141 Essen, Germany

**Keywords:** Climate-change ecology, Soil microbiology, Carbon cycle

Correction to: *Nature Communications* 10.1038/s41467-020-15812-w, published online 20 April 2020.

The original version of this Article contained errors in Figure 2b, where the y-axis was labelled “cm” when the unit should have been “mm”, and the y-axis error bars should have been omitted. These have been corrected in both the PDF and HTML versions of the Article.

