# Supplementary material for: Cable bacteria reduce methane emissions from rice-vegetated soils
Source: Nat Commun. 2020 Apr 20;11:1878. doi: 10.1038/s41467-020-15812-w (PMC7171082; doi:10.1038/s41467-020-15812-w)
Supplement: Supplementary file 1 — Supplementary Information [file 41467_2020_15812_MOESM1_ESM.pdf]

Supplementary Information for

Cable bacteria reduce methane emissions from rice-vegetated soils

by Scholz et al.

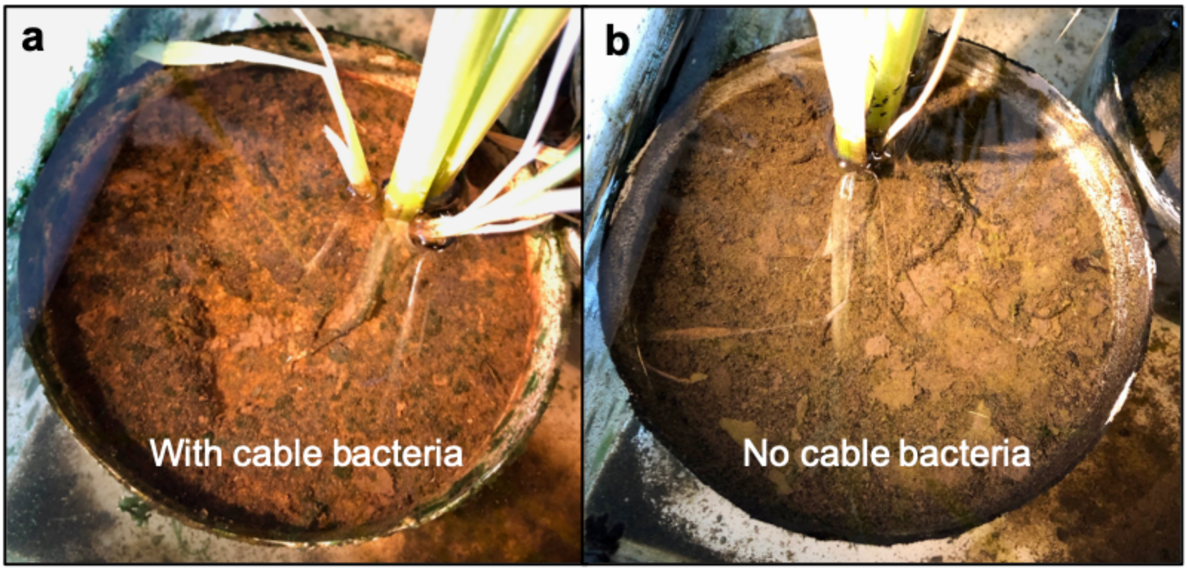

Supplementary Fig. 1 Images of the soil surface. Rice pot with cable bacteria **(a)** and without cable bacteria **(b)**. The above-ground biomass of single plants grown in soil with cable bacteria was 3.4 ± 0.3 g dry weight and in soil without cable bacteria 3.6 ± 0.3 g dry weight (mean ± standard error of the mean, n=4).

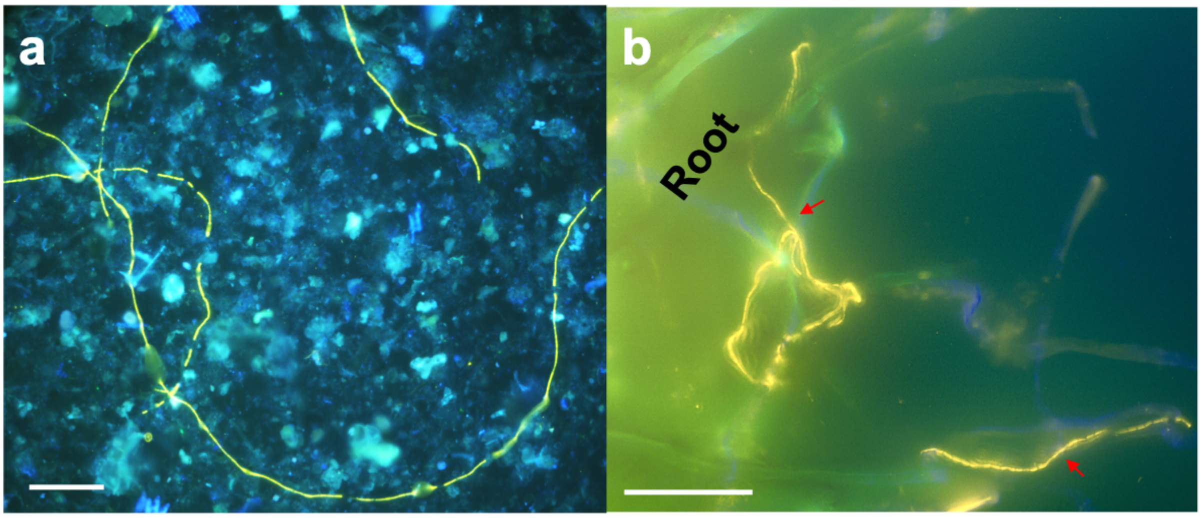

Supplementary Fig. 2 Cable bacteria in inoculated rice soils. a, Cable bacteria in the bulk soil. **b**, Cable bacteria in close contact with a rice root. Images from FISH hybridized with probe DSB706 specific for *Desulfobulbaceae* labeled with Cy3 (red) and probe EUB-MIX targeting most bacteria labeled with Atto-488 (green) are overlaid with images from DAPI staining (blue). Scale bars, 50 µm.

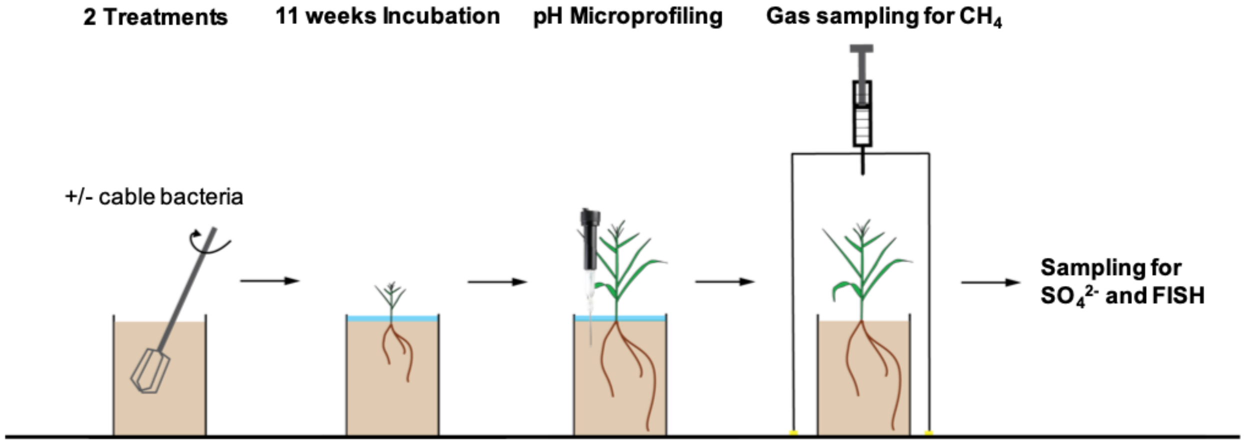

Supplementary Fig. 3 The experimental design.
