# Supplementary material for: Cable bacteria reduce methane emissions from rice-vegetated soils
Source: Nat Commun. 2020 Apr 20;11:1878. doi: 10.1038/s41467-020-15812-w (PMC7171082; doi:10.1038/s41467-020-15812-w)
Supplement: Supplementary file 3 — Reporting Summary [file 41467_2020_15812_MOESM3_ESM.pdf]

nature research

Corresponding author(s): Vincent Valentin Scholz

Last updated by author(s): Mar 5, 2020

Reporting Summary

Nature Research wishes to improve the reproducibility of the work that we publish. This form provides structure for consistency and transparency in reporting. For further information on Nature Research policies, see Authors & Referees and the Editorial Policy Checklist.

Statistics

For all statistical analyses, confirm that the following items are present in the figure legend, table legend, main text, or Methods section.

n/a	Confirmed	
☐	☒	The exact sample size (*n*) for each experimental group/condition, given as a discrete number and unit of measurement
☐	☒	A statement on whether measurements were taken from distinct samples or whether the same sample was measured repeatedly
☐	☒	The statistical test(s) used AND whether they are one- or two-sided<br>*Only common tests should be described solely by name; describe more complex techniques in the Methods section.*
☒	☐	A description of all covariates tested
☒	☐	A description of any assumptions or corrections, such as tests of normality and adjustment for multiple comparisons
☐	☒	A full description of the statistical parameters including central tendency (e.g. means) or other basic estimates (e.g. regression coefficient) AND variation (e.g. standard deviation) or associated estimates of uncertainty (e.g. confidence intervals)
☐	☒	For null hypothesis testing, the test statistic (e.g. *F*, *t*, *r*) with confidence intervals, effect sizes, degrees of freedom and *P* value noted<br>*Give P values as exact values whenever suitable.*
☒	☐	For Bayesian analysis, information on the choice of priors and Markov chain Monte Carlo settings
☒	☐	For hierarchical and complex designs, identification of the appropriate level for tests and full reporting of outcomes
☒	☐	Estimates of effect sizes (e.g. Cohen's *d*, Pearson's *r*), indicating how they were calculated

Our web collection on statistics for biologists contains articles on many of the points above.

Software and code

Policy information about availability of computer code

Data collection	For FISH analysis NIS-Elements (Version 4.50; Nikon Instruments Inc., United States), for methane measurement the software PeakSimple (Version 4.44 Alan Hetherington, SRI Inc., United States), for sulfate measurement the software Chromeleon (Version 6.80, Dionex Corporation, United States) and for pH microprofiling SensorTracePro (Unisense, Denmark) was used.
Data analysis	For data analysis the same softwares as used for data collection and in addition Microsoft Excel (Microsoft Corporation, United States) were used.

For manuscripts utilizing custom algorithms or software that are central to the research but not yet described in published literature, software must be made available to editors/reviewers. We strongly encourage code deposition in a community repository (e.g. GitHub). See the Nature Research guidelines for submitting code & software for further information.

Data

Policy information about availability of data

All manuscripts must include a data availability statement. This statement should provide the following information, where applicable:

- Accession codes, unique identifiers, or web links for publicly available datasets
- A list of figures that have associated raw data
- A description of any restrictions on data availability

The raw data generated in this study is available from the corresponding author upon request. Fig. 1-3 have associated data.

Field-specific reporting

Please select the one below that is the best fit for your research. If you are not sure, read the appropriate sections before making your selection.

☐ Life sciences ☐ Behavioural & social sciences ☒ Ecological, evolutionary & environmental sciences

For a reference copy of the document with all sections, see nature.com/documents/nr-reporting-summary-flat.pdf

Ecological, evolutionary & environmental sciences study design

All studies must disclose on these points even when the disclosure is negative.

Study description	Four replicates of rice pots from each of the two treatments (with and without cable bacteria) were incubated in containers with tap water.
Research sample	Rice plants were grown in pots containing wetland soil and cow dung. Before the rice plants were planted, half of the pots were inoculated with cable bacteria using the cable bacteria enrichment culture Ca. Electronema sp. GS (Kjeldsen, K. U. et al. On the evolution and physiology of cable bacteria. PNAS 116, 19116-19125 (2019)).
Sampling strategy	For FISH and sulfate analysis, the soil was sampled down to 4 cm depth. Due to practical difficulties and excessive root growth at the bottom of the pots, deeper soil layers could not be retrieved. After inverting the rice pot and carefully opening the soil matrix, roots were randomly chosen and sampled including the adhering soil for FISH analysis. For pH microprofiling, the soil depth of 7.2 mm was chosen to be sufficient to determine a difference of pH between the treatments. Deeper profiling would risk that the sensor tip breaks due to collision with roots. For the determination of methane emissions, two chambers with one pot from each treatment were incubated at the same time.
Data collection	The data was collected and has been stored digitally by Vincent Valentin Scholz.
Timing and spatial scale	The pot experiment was set-up on the 29.04.2019 and sampled during the time between the 14.07.19 and the 17.07.2019. The incubation time is stated as 11 weeks.
Data exclusions	pH depth profiles were depth-corrected in Microsoft Excel (Microsoft Corporation, United States) because the soil-water interface could not be precisely adjusted as starting and reference point with the micromanipulator. Therefore only depth-corrected datapoints in the same depth of each replicate are displayed in Fig. 2b.
Reproducibility	Reproducibility was achieved through replication (n=4) per treatment.
Randomization	The two gas collection chambers were randomly assigned to rice pots in each of the four replicate measurements. One rice pot of each treatment was simultaneously analyzed for methane.
Blinding	Blinding of samples was not possible as one rice pot of each treatment was simultaneously analyzed for methane and one rice pot after the other for all other measurements.

Did the study involve field work? ☐ Yes ☒ No

Reporting for specific materials, systems and methods

We require information from authors about some types of materials, experimental systems and methods used in many studies. Here, indicate whether each material, system or method listed is relevant to your study. If you are not sure if a list item applies to your research, read the appropriate section before selecting a response.

Materials & experimental systems

n/a	Involved in the study	
☒	☐	Antibodies
☒	☐	Eukaryotic cell lines
☒	☐	Palaeontology
☐	☒	Animals and other organisms
☒	☐	Human research participants
☒	☐	Clinical data

Methods

n/a	Involved in the study	
☒	☐	ChIP-seq
☒	☐	Flow cytometry
☒	☐	MRI-based neuroimaging

Animals and other organisms

Policy information about studies involving animals; ARRIVE guidelines recommended for reporting animal research

Laboratory animals	The study did not involve laboratory animals.

Wild animals	The study did not involve wild animals.
Field-collected samples	The study did not involve samples collected in the field.
Ethics oversight	No ethical approval or guidance was required as the study involved non-pathogenic microorganisms

Note that full information on the approval of the study protocol must also be provided in the manuscript.
